# Strengthen causal models for better conservation outcomes for human well-being

**DOI:** 10.1371/journal.pone.0230495

**Published:** 2020-03-20

**Authors:** Samantha H. Cheng, Madeleine C. McKinnon, Yuta J. Masuda, Ruth Garside, Kelly W. Jones, Daniel C. Miller, Andrew S. Pullin, William J. Sutherland, Caitlin Augustin, David A. Gill, Supin Wongbusarakum, David Wilkie

**Affiliations:** 1 National Center for Ecological Analysis and Synthesis, University of California-Santa Barbara, Santa Barbara, CA, United States of America; 2 Center for Biodiversity and Conservation, American Museum of Natural History, New York, CA, United States of America; 3 Bright Impact, San Francisco, CA, United States of America; 4 Global Science, The Nature Conservancy, Seattle, WA, United States of America; 5 European Centre for Environment and Human Health, Truro, England, United Kingdom; 6 Colorado State University, Fort Collins, CO, United States of America; 7 University of Illinois, Urbana-Champaign, IL, United States of America; 8 Center for Evidence-based Conservation, Bangor University, Bangor, Wales, United Kingdom; 9 Department of Zoology, University of Cambridge, Cambridge, England, United Kingdom; 10 DataKind, New York, NY, United States of America; 11 Moore Center for Science, Conservation International, Arlington, VA, United States of America; 12 Environmental Science and Policy, George Mason University, Fairfax, Virginia, United States of America; 13 Duke University Marine Laboratory, Nicholas School of the Environment, Duke University, Beaufort, North Carolina, United States of America; 14 National Oceanic and Atmospheric Administration, Honolulu, HI, United States of America; 15 Wildlife Conservation Society, Bronx, NY, United States of America; Kyoto University, JAPAN

## Abstract

**Background:**

Understanding how the conservation of nature can lead to improvement in human conditions is a research area with significant growth and attention. Progress towards effective conservation requires understanding mechanisms for achieving impact within complex social-ecological systems. Causal models are useful tools for defining plausible pathways from conservation actions to impacts on nature and people. Evaluating the potential of different strategies for delivering co-benefits for nature and people will require the use and testing of clear causal models that explicitly define the logic and assumptions behind cause and effect relationships.

**Objectives and methods:**

In this study, we outline criteria for credible causal models and systematically evaluated their use in a broad base of literature (~1,000 peer-reviewed and grey literature articles from a published systematic evidence map) on links between nature-based conservation actions and human well-being impacts.

**Results:**

Out of 1,027 publications identified, only ~20% of articles used any type of causal models to guide their work, and only 14 total articles fulfilled all criteria for credibility. Articles rarely tested the validity of models with empirical data.

**Implications:**

Not using causal models risks poorly defined strategies, misunderstanding of potential mechanisms for affecting change, inefficient use of resources, and focusing on implausible efforts for achieving sustainability.

## Introduction

Increasingly, nature conservation is seen as a viable global strategy for simultaneously improving human well-being and achieving environmental sustainability [1, 2]. These policies are predicated on the assumption that human well-being challenges can be addressed by maintaining or improving environmental conditions, particularly through the provisioning of natural resources and ecosystem services [3]. For example, nature conservation policies and practices include: protecting mangroves to reduce the impacts from tsunamis [4, 5]; urban tree planting to combat the negative effects of air pollution [6] and heat island effects [7]; and curbing schistosomiasis by reintroducing native river prawns [8]. But theory and evidence on whether, how, and to what extent these nature-based conservation interventions affect human well-being is relatively nascent [9], raising questions about the risks, requisite resources, and ultimately the role of conservation in achieving objectives across complex social-ecological systems. As such, in order to design and implement effective solutions, better and greater understanding of where, when, and which policies and actions lead to desired outcomes, is needed. In short, we need a clear understanding of the *causality* of nature conservation interventions in relation to intended outcomes for human well-being.

Designing effective conservation increasingly requires thinking about how interventions are situated within linked social and ecological systems where pathways are often interconnected and synergistic [10, 11]. Thus, in the face of complexity, there is a need for using more systems-based approaches that clearly articulate how components within social-ecological systems are connected [12]. This is especially important if we want to understand how changing one component can lead to cascading effects throughout a system, while also mitigating unintended consequences. Thus, achieving sustainability requires understanding complex patterns of cause and effect that are often not linear, but occur in feedback loops with multiple externalities and enabling conditions–particularly in the case of links between the ecosystems and well-being of people [13].

Across numerous disciplines, *causal models* have emerged as a critical tool for explicitly describing hypotheses of how cause and effect occur in complex systems. Causal models, as a whole, detail the logic and assumptions around how a series of interdependent steps will lead to intended outcomes. Causal models are not a new concept. In its simplest form, it is a type of hypothesis, but also often described as conceptual or theoretical models and frameworks. Examples include complex causation frameworks in political science [14], process models in engineering [15], structural model evaluations in business [16], and theories of change [17] and results chains [18] in development and conservation. In essence, these *causal models* all ask–how is an intervention or suite of interventions assumed to lead to desired outcomes? Based on underlying theory, these models explicitly describe mechanisms necessary to achieve goals, articulate assumptions, and clarify interdependencies between actions and objectives. Further, they provide a structured framework for defining and examining these relationships in both simulated and real-world contexts.

A causal model differs from two other types of models, methodological and conceptual models, which are commonly used to make sense of relationships and linkages within a system. A methodological model is used to *test* for causality. For example, difference in differences (DID) estimation (Yi = α + βTi + γti + δ (Ti · ti) + εi) is an example of a formulaic model, which is used to estimate the effect of a specific intervention or treatment (such as a passage of law, enactment of policy, or large-scale program implementation) by comparing the changes in outcomes over time between a population that is enrolled in a program (the intervention group) and a population that is not (the control group) [19, 20]. This method is used to mimic an experimental design by obtaining an appropriate counterfactual from observational data in order to estimate a causal effect. Conceptual models identify and characterize the existing conditions and key drivers that affect the current status of social or environmental variables within a system. They usually visually portray the relationships among the different factors within a situation analysis [21, 22]. We clarify here that our study speaks to causal models specifically, versus the methods used to test for causal relationships, or broader models which describe entire socio-ecological or political systems.

A significant body of research has strongly argued for the utility of using causal model diagrams [23–25], citing, for instance, their usefulness for describing assumptions and hypotheses [26, 27], designing monitoring and evaluation plans [28], and explaining complex topics to lay audiences [29]. For example, clear articulation of the steps required to get from an intervention to a desired outcome can inform evaluation design by outlining key checkpoints and indicators to measure progress throughout a program life cycle [27]. Causal models are increasingly required by funders and used by implementing agencies and organizations interested in advancing sustainability goals. In the past decade, The Nature Conservancy [30], United States Environmental Protection Agency [31], Conservation International [32], Britain’s Department for International Development [27], the United States Agency for International Development [33], and others have emphasized the need for, and utility of, causal models. Using causal models can support critical thinking about how and why change can happen throughout a program life cycle, enabling more responsive and adaptive planning in complex situations. While the attention is welcome, little work has evaluated how these causal models are actually used in practice and research in conservation.

We aim to address this knowledge gap by examining over 1,000 scientific research articles on the linkages between conservation and human well-being outcomes from a systematic evidence map [34]. This corpus of literature is representative of extant approaches to evaluating the effect of conservation interventions, and thus can be illustrative of the extent and mode of application of causal models in the field.

### Developing criteria for assessing causal models

Here, we take a broad, multi-disciplinary view of causal models and thus draw on available guidance and representative models from a diversity of sources ([17, 18, 21, 22, 27, 28, 35], including the Center for Theory of Change [36]) to develop three comprehensive criteria for assessing the credibility of employed causal models in conservation. We define a *credible causal model* as one that comprehensively articulates a causal pathway between actions, intermediate outputs, and a set of resultant outcomes, and is explicit about key assumptions and mechanisms between steps. Below, we explain each criterion and our rationale for their inclusion in our assessment rubric:

**Criterion 1:** *Does it illustrate and describe a causal process of change*? For example, description of models must describe a cause and effect relationship between an action X to outcome Y. It cannot simply indicate a link between two elements.

As this study focuses on causal models (versus methodological or conceptual models), explicit description of a cause and effect relationship–for example, identifying actions and outcomes–is required. Many of the causal models we surveyed to define these criteria often included unlinked elements, for example, to highlight important enabling conditions or different states. However, all models consistently emphasized the importance of clearly identifying which components were interlinked and the direction of that link (e.g. cause vs. effect).

**Criterion 2:** *Does it clearly outline a* ***comprehensive*** *set of intermediary steps and/or necessary pre-conditions or factors for the long-term outcome(s) to be achieved*?

Working in complex scenarios requires in-depth thinking around the different pathways through which change and impact can occur, as well as consideration of how individual contexts can influence performance of an intervention. Thus, explicit and comprehensive outlining of the steps required to get from an intervention to an outcome lends greater clarity around what we expect to happen (e.g. a hypothesis), supports our ability to test its validity [37], as well as determine factors that contribute to unexpected outcomes. This can provide more detailed and practical information around how to improve and adapt conservation interventions versus just knowing if something worked or not. For example, models meeting this criterion would describe the entire set of required enabling conditions. Where it is logically reasonable (i.e. depending on where along the causal pathway the study focuses), models meeting this criterion would also describe the entire hypothesized set of intermediate outputs that are required prior to achieving desired outcomes (e.g. chain of outcomes).

**Criterion 3:** *Is the model* ***explicit*** *in outlining assumptions and hypotheses about how an action influences a series of intermediary outcomes that lead to desired outcomes*? For example, models meeting this criterion would detail *how* doing X action will lead to Y outcome because of Z, assuming A, B, and C conditions hold true.

Model approaches we reviewed consistently emphasize the importance of transparent and sufficient articulation of assumptions about how and why interventions lead to desired outcomes. Assumptions are often framed in program theory and program design as the “things that we believe to be true” and reflect the beliefs and perspectives of whomever created the model in question [36]. They can be thought of as the process that leads from one change to another–for example, the theory that increased environmental education will lead to increase in pro-environmental behaviors is often based on the assumption that individuals make decisions based on information they receive from an educational program [38]. Knowing what these assumptions are helps facilitate more deliberate choices in intervention design depending on if they are context-appropriate. As there will always be multiple perspectives to how and why change will occur, clarity on assumptions is critical for readers appropriately interpret findings in their own contexts.

## Methods

We explored if and how causal models are used in conservation by assessing a set of 1,027 articles derived from a previously published, peer-reviewed systematic map of evidence linking conservation effects to human well-being outcomes [34, 35]. Systematic maps are a thematic collections of empirical research studies and systematic reviews within a sector that maps the distribution and occurrence of existing evidence using a framework of policy-relevant interventions and outcomes [39]. Systematic maps are increasingly being employed in the environmental management and conservation sector to provide clear, synthesized assessments of where critical knowledge gaps exist to guide future research prioritization and illuminate areas of uncertainty [9]. This systematic map focused on non-OECD countries and included a broad range of interventions, study designs (ranging from non-experimental to experimental designs with quantitative and qualitative data), and human well-being outcomes (Table 1). Included articles were compiled using a Boolean search string to query peer-reviewed literature databases and grey literature sources per McKinnon et al. 2016. The systematic map was conducted following standards and guidelines from the Collaboration for Environmental Evidence.

**Table 1:** Scope covered in the conservation literature dataset used in this analysis (derived from McKinnon et al. 2016).

**Relevant populations**	The study focuses on the well-being of discrete individuals, households or communities, or nation states living in non-OECD countries
**Relevant interventions**	The study involves establishment, adoption, implementation or refinement of a program or policy that regulates, protects or manages biodiversity and natural ecosystems through *in situ* activities as categorized by the IUCN Conservation Measures Partnership (CMP) Classification of Direct Actions [40]
**Relevant comparators**	The study involves empirical quantitative and/or qualitative measurement of direct or indirect effects of a policy or program.
**Relevant outcomes**	The study measures or observes effects on one or more domains of human well-being categorized as follows: Economic Living Standards, Material Living Standards, Governance and Empowerment, Education and Capacity Building, Health, Subjective well-being, Security and Safety, Culture and Spirituality, Social Relations, Freedom of Choice and Action
**Relevant models**	Use of a conceptual and/or modular model to express causal thinking

### Data coding strategy

**Fig 1:**
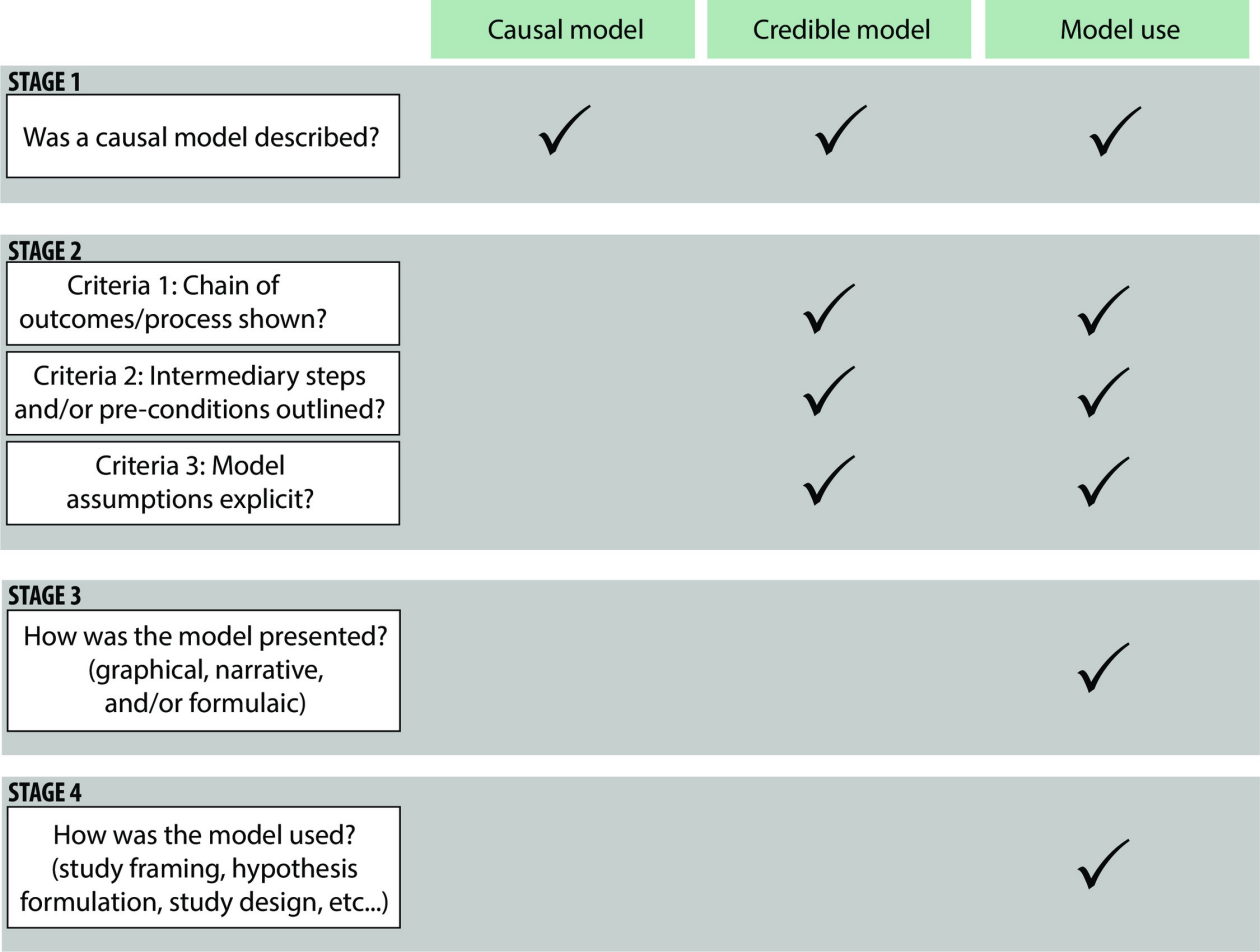
Coding scheme for assessing causal models, credible causal models, and use.

We use this evidence base to draw additional inference on causal model occurrence and use by further examining included studies regard their use of causal models. All articles were screened and examined in four stages (Fig 1, Table 2). We screened articles for inclusion based on if they employed any type of conceptual or modular model to capture causal thinking (see Stage 1 below) (Table 2). Studies were screened by two reviewers, and conflicts were discussed and resolved, with a third reviewer if needed. Included studies were then coded using a standard data extraction questionnaire to capture model characteristics, credible causal model criteria (see Stage 2 below), and information on how the models are presented (see Stage 3) and how they are used within the context of the study (see Stage 4) (S1 Table, Table 3). Bibliographic, intervention type, outcome type, and study design type information were drawn from the original systematic map dataset.

**Table 2:** Summary of screening and coding strategy.

Stage	Objective	Assessment criteria
Stage 1	*To explore the extent that causal thinking is employed in conservation*, *we considered any attempt to attribute causality using modular or conceptual models*.	Did the article employ any kind of conceptual model, whether narrative, graphical, or mathematical, to understand and explain causal relationships? Studies where the causal model was unclear or had to be inferred by the reviewer (i.e. assuming the authors were using the model to understand causality rather than it being explicitly stated) were coded as not employing a model.
Stage 2	*We then coded all included articles from stage 1 on the credibility of these causal models based on criteria 1–3*.	If the article did employ an attempted causal model (graphical, narrative or formulaic), was it credible? We provide examples of scored articles with credible and non-credible causal models in Table 3.
Stage 3	*We then coded all included articles from stage 1 for how models were depicted (e*.*g*. *models can be depicted using formulas*, *text*, *boxes and arrows*, *flow diagrams*, *visual diagrams*, *and/or a combination thereof)*	An important component of a credible causal model is the clarity by which it communicated. This is particularly relevant for conservation—being a multidisciplinary space—terminology and language used to describe change pathways varies across disciplines, as well as language and cultural boundaries [37, 38]. Thus, different approaches to describing models (e.g. narrative and non-narrative) can be potentially useful for facilitating understandings across different disciplines and groups. For example, visual graphics are often used to summarize and depict patterns and trends in data for broad communication of scientific findings, frameworks, and theories [39, 40]. Similarly, formulaic representations are also useful for distilling relationships and linkages into an intuitive format. Narrative descriptions can add clarity to otherwise more simplified non-narrative notations.
Stage 4	*All articles from Stage 3 were then closely examined to determine how these models were employed*.	If the model employed is graphical, causal, and credible, how was the model used? For example, models could be used to frame the study, aid in formulating a hypothesis (or hypotheses), guide the study design, choose indicators to measure outcomes, and/or analyse the results

**Table 3:** Paired example of credible (bold) and non-credible causal models from the conservation and human well-being evidence base.

ID	Intervention	Outcomes	Criterion 1: *Does it illustrate a causal change process or chain of outcomes*?	Criterion 2: *Does it outline a comprehensive set of intermediary steps and/or pre-conditions*?	Criterion 3: *Is the model explicit in outlining assumptions and hypotheses about cause & effect*?	Stage 3: How are the models presented?
811	Protected areas, law & policy	Governance & empowerment, social relations	No (illustrates organizational linkages but not cause and effect relationships)	Yes (illustrates strength of organizational linkages required for empowerment)	No (does not explain how organizational linkages lead to empowerment)	Graphical depiction of linkages
**827**	**Protected areas, livelihood alternatives**	**Economic living standards, material living standards**	**Yes (illustrates how singular and repeat disturbances has impacts on defined outcomes)**	**Yes (describes series of cause and effect chains)**	**Yes (explicitly states assumptions about behavior of communities in context of disturbances)**	**Graphical depiction of concept**
106	Law & policy, resource management	Economic living standards, education, health, material living standards, social relations	No (illustrates links between livelihood components but not causal relationships)	Yes (illustrates livelihood preconditions)	No (Is not explicit about how livelihood components are impacted by actions)	Graphical depiction of linkages between components
**1052**	**Livelihood alternatives**	**Economic living standards, social relations, culture**	**Yes (illustrates set of steps from local participation to increased conservation)**	**Yes (outlines intermediate outputs and parallel pathways)**	**Yes (provides explicit assumptions and hypotheses regarding links between actions and intermediate outputs)**	**Graphical depiction of cause and effect**

All studies were systematically screened and coded using the standard data extraction questionnaire by a team of reviewers and results were cross-checked between at least two reviewers for consistency. Using the generalized linear model (glm) function in the ‘base’ package in R [41], we conducted a binomial regression to examine the impact of two independent variables (impact assessments and publication year) on the dependent variable (use of any causal models) until the model converged (~5000 iterations). As there were few studies with *credible causal models* (n = 14), we qualitatively describe the characteristics of these.

## Results

**Fig 2:**
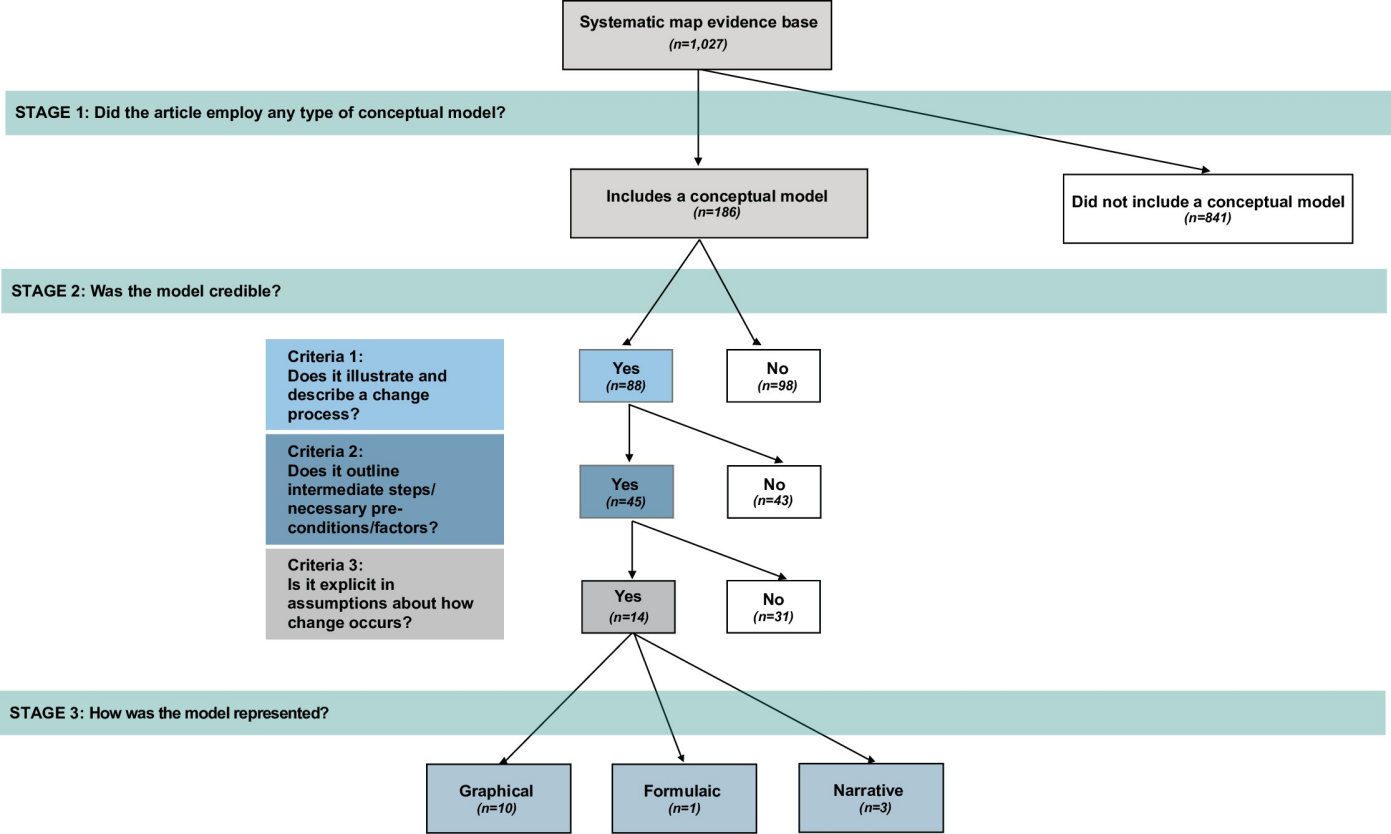
Proportion of evidence base that met criteria for credible causal models.

We found that the vast majority of the examined evidence base neglects to document in any fashion (graphical, narrative, and/or formulaic), the underlying mechanisms about how the studied conservation intervention is predicted to affect human well-being outcomes. Only a fifth (18.1%; n = 186) of the evidence base employed any type of causal model (graphical, narrative, and/or formulaic) to frame the study, choose indicators and design analyses, and/or evaluate and interpret results (Fig 2). Only ~1% of the total dataset (n = 14) fulfilled all criteria for credibility (Fig 2, S1 Table, S1 File). Most of these studies were presented graphically (n = 10) while 3 were presented narratively only and 1 in formulaic notation. Examining all studies that employed some type of causal model, most (53.7%, n = 100) depicted their model graphically, while others solely described their model narratively (n = 72) or with formulaic representation (n = 12). (S1 Table). We found that most examined models failed criteria 2 and 3 for credibility (56.5% and 75.8% respectively).

For each year after 1990, the odds of finding an article employing any type of causal model increased by ~3% (odds ratio = 1.099, p<0.01), indicating increased use over time. On the other hand, more empirically robust studies (i.e. those that attempted to evaluate effect size using a counterfactual), were not significantly more likely to employ a causal model at all (odds ratio = 0.788, p>0.1). Of the all the articles that measured impacts using a before/after or with/without comparison in the evidence base (n = 86)– 11 described any type of model, with only 2 of those fulfilling criteria 1–3 for credibility. This demonstrates that of the studies which we *expect* to clearly articulate how and why an intervention may lead to outcomes, the use of credible causal models (of any form) is remarkably low.

Of the 14 studies that employed credible causal models, all examined the impact of conservation interventions in forest ecosystems. Studies mostly focused on the impacts of alternative livelihood projects (n = 5), protected areas (n = 4) and resource management (n = 3). In terms of outcomes, almost all studies measured economic living standards (n = 13). Only two articles utilized an empirically robust study design (i.e. counterfactual). Most studies used causal models to frame the study, but rarely tested or analysed the relationships indicated in the models (S2 Table). Eight of the credible causal models were novel while others used previously established models or modifications thereof. For example, Salafsky et al. (2001) [42] used a previously published model [43] whereas Pegas et al. (2013) [44] modified an existing model [45] (S2 Table). Of the studies that used any type of model (credible or not) (n = 186), the majority either were insufficient (e.g. highlighted components, not pathways) or descriptive narratives (e.g. simply stating “this framework describes various assets and how they influence environment and human well-being”) that are vulnerable to subjective interpretation.

## Discussion

Causal models are increasingly highlighted as a valuable tool for illustrating and understanding relationships and interactions between interventions, outcomes, and impacts; yet our analysis identified few documented models within a large, recent, and relevant evidence base. This gap might be pragmatic given publication constraints, or possibly more symptomatic of broader concerns around a deficit in critical thinking, or a lack of incentives for comprehensive reporting.

The lack of documented causal models may be due to a number of pragmatic factors, including journal constraints on content or length, lack of standards around consistent reporting, and/or low visibility of causal models (e.g. not explicitly stating that they were used and whether they may be described in the paper or supplementary materials). All of these factors may result in a low reporting of causal models, even if they were used. The multidisciplinary nature of conservation means standardized reporting of meta-data from evaluation studies and impact research frequently varies across fields [46]. This problem is not unique to conservation—recent studies highlight a concerted need for adopting meta-data standards (e.g. Dublin Core [47]) for ensuring that published research is easier to find for efforts such as systematic reviews and meta-analyses [48]. While efforts to standardize reporting in conservation, are growing, for example through efforts like establishing a common lexicon for conservation topics [40], these standards are not widely adopted by publishers and journals and the onus remains on individual authors to use them.

A lack of documented causal models may also partially reflect limitations of our evidence base [34]. Our strategy for compiling the evidence base was intended to be comprehensive (i.e. capturing the breadth of topics relevant to links between conservation and human well-being) but not exhaustive (i.e. not attempting to capture every extant published study). For example, while the methods used to generate the systematic map attempted to comprehensively capture evidence from grey literature (unpublished literature) sources, it recognizes that some sources and reports may have been missed (see [34]). While the evidence base interrogated was not intended to be exhaustive, we would still expect given the complexity of understanding linkages between conservation and well-being and the breadth of the topic at hand, that this topic would be a priority area for considering causal relationships.

We found that documented credible causal models tended to be presented graphically, occasionally complemented by narrative descriptions. For example, models were often depicted using boxes and arrows, flow diagrams, and/or a combination of visual graphics and text. Amongst the authors of this study, we found that this type of visual depiction was very useful for us to clearly understand the components and pathways that were being described and investigated. Overall, around half of the articles from the evidence base employing any type of conceptual model used a graphical depiction (n = 100). Using graphical depictions is particularly relevant for conservation—being a multidisciplinary space–as the terminology and language used to describe change pathways varies across disciplines, as well as language and cultural boundaries [46, 49]. Thus, non-narrative approaches to describing models could be potentially useful for facilitating broader understandings of pathways of change and system dynamics, and pattern discovery, across different disciplines and groups [23]. For example, visual graphics are often used to summarize and depict patterns and trends in data for broad communication of scientific findings, frameworks, and theories [50, 51]. Similarly, while formulaic representations are also useful for distilling relationships and linkages into an intuitive format, they can also be limited given it is more difficult to incorporate explicit details on assumptions, conditions, and linked pathways. Thus, visual depictions of how interventions can lead to desired outcomes, such as flow diagrams or matrices, can be more accessible to a broader audience [18, 23]. However, we do recognize that these depictions are not universally accessible, for example, for the visually impaired. Thus, in order to be useful, visual depictions should be accompanied by a detailed narrative description.

Conceptual models were often employed to illustrate frameworks for categorizing outcomes and sets of enabling conditions related to the studied intervention, as opposed to describing an explicit causal relationship (S1 Table). For example, a number of articles (e.g. [52–55]) used frameworks to categorize different livelihoods assets/resources deriving from the Sustainable Livelihoods Framework [56] across different socio-economic groups within a conservation intervention (e.g. a protected area, species protection program).

Of the studies that did utilize credible causal models (n = 14), we find that the methodologies employed to test for causality were quite varied, and articles often did not employ robust methodologies ((i.e. using before/after and/or with/without counterfactuals to attribute observed outcomes to the presence of a conservation intervention) for either quantitative nor qualitative data. Conversely, out of all the articles in the evidence base that employed robust quantitative methodologies (n = 67 of 1,027 articles, McKinnon et al. 2016)–very few of them defined any type of causal model at all (4 out of 11 studies), much less a credible one (2 out of 11 studies).

### Implications for conservation research and practice

There are three obvious risks or consequences in not using credible causal models (both graphical and in other forms) for conservation research, and, more broadly, decision-making.

First, without adequate explanation or theory of how interventions are likely to achieve results, we risk making assumptions that are, at best, unsupported and at worse, implausible [57, 58]. Thus, to test whether existing assumptions around causal relationships are valid, models must detail *how* and *why* activities are thought to lead to particular outcomes [59]. If the intent is to apply research insights to inform conservation practice, these assumptions and the underlying theory that supports them, need to be clearly articulated so as to understand whether study findings are reliable, much less applicable to different contexts.

Second, while we find many graphical depictions of causal models, a significant portion of the evidence base only described their models narratively. While narrative models are common, it can have implications for the extent of external validity of published research–as it constrains the ability of others to replicate studies or test specific hypotheses in different contexts. This study found graphical models complemented with a narrative description improved the ability of reviewers to understand and interpret the causal models in use. Graphical models are particularly helpful in providing a cross-cutting, intuitive framework to align planning across multiple disciplines, and contexts as well as cross-project learning and adaptation [18]. This is particularly critical as models should be interpretable across disciplinary, sectoral, and cultural boundaries in order to facilitate collaboration and communication in global initiatives to achieve sustainable landscapes at scale.

Finally, without clearly articulated models, it can be difficult to fully capture and understand relationships between variables in complex systems, including identifying where interdependencies, feedbacks, trade-offs, and unintended consequences may occur or appear. This can make it difficult to isolate factors that affect the magnitude, attribution, and timing of observed results, particularly when analysing empirical data on impacts. For example, without defining explicitly how we think X is connected to outcomes Y and Z, it will be difficult to appropriately test for this relationship, much less see when there are potential feedbacks between X and Y or trade-offs between Y and Z. Moreover, typically a number of intermediate outcomes must be in place in order to achieve longer-term outcomes that conservation aims for–for example, recovered ecosystem functions and decreased human poverty. Thus, without clear and detailed mapping of a hypothesized pathway to outcomes, it can be difficult to determine where problems occur and identify intervention points for adaptive management [18]. This is particularly important as the dynamic nature of conservation challenges demand adaptive, responsive science and policies [60].

These risks are particularly problematic for making progress towards evidence-informed conservation practice and policy and bridging the gap between science and action. While the use of causal models (such as theory of change and results chains) is becoming a standard of practice in conservation and development organizations [13, 27, 61], the formulation and updating of these models will need to be informed by the breadth of existing empirical evidence, which continues to grow exponentially, particularly in environmental disciplines [62]. Applying insights from this growing body of work to these models will remain difficult if it is not clear how individual findings are relevant or whether they are appropriate for the model in question [63].

Recently, guidelines for generating causal models for conservation are under development in order to facilitate a shared, cross-cutting evidence base with a common ontology across studies and disciplines, e.g. the Conservation Actions and Measures Library (CAML, http://cmp-openstandards.org/tools/caml/). Additionally, there has been a movement to develop and use *reference* causal models for sustainability by major funders and organizations (e.g. USAID, USFWS, Bridge Collaborative, Conservation Measures Partnership) to inform strategic priorities, activities, and evaluation of impact. These efforts can help practitioners and policymakers avoid repeating errors and for donors to compare across streams of work, using a common framework.

We recognize that not all research articles require causal models. However, the risks of not using a credible causal model in research that *intends* to evaluate causal impact of conservation, particularly for the purposes of informing conservation practice, are high. Thus, to facilitate progress in this area, we outline the following recommendations:

- *Promote use and reporting*: We suggest that journals strongly encourage authors to include articulated causal models in submissions of empirical evaluations of interventions. Doing so will address low reporting of causal models and facilitate both greater use and transparency of models in literature.
- *Consider using visual*, *graphical depictions of causal models*: We particularly encourage authors to consider communicating their models using graphical notations along with any other narrative or formulaic descriptions. Doing so will improve transparency and communication of articulated causal linkages and hypotheses around system connections.
- *Apply standards of practice*: We encourage the conservation research and practice community to establish a minimum set of requirements for causal models to ensure transparency, replicability, credibility and integration into project design, funding requirements, and business processes. By doing so, we believe that this will help establish and foster the uptake of a new “gold standard” of practice. For example, for new proposed work, funders should require use of models and description of *how project implementers will use the models* to foster a standard of practice in making logical and well-supported value propositions. Among researchers and practitioners, we suggest that causal models should be more consistently integrated into project design and reporting. This is likely to require training and capacity-building.
- *Increase visibility and transparency of models*: We encourage researchers and practitioners who are developing or have developed causal models to contribute to new or existing repositories in order to increase both model visibility and transparency. For example, the Conservation Actions and Measures Library hosts a repository for results chains and causal models that could be a good option to start. By making models openly available, this can make thinking more intelligible and explicit to a more diverse audience in an interdisciplinary context. We especially encourage increased visibility in order to help build an active community of practice around creating and validating causal models in conservation and sustainability writ large.

Achieving global sustainability requires developing sound interdisciplinary theories that will facilitate collaboration amongst diverse audiences and minimize misinterpretation. Consistent–and standardized—use of causal models can advance progress towards understanding how conservation impacts social-ecological systems by bringing subfields together to more holistically examine how impact occurs across entire systems.

## Supporting information

S1 Checklist (DOC)

S1 Table (XLSX)

S2 Table (XLSX)

S1 File (DOC)
