# Supplementary material for: Strengthen causal models for better conservation outcomes for human well-being
Source: PLoS One. 2020 Mar 20;15(3):e0230495. doi: 10.1371/journal.pone.0230495 (PMC7083336; doi:10.1371/journal.pone.0230495)
Supplement: S1 File — (DOC) [file pone.0230495.s004.doc]

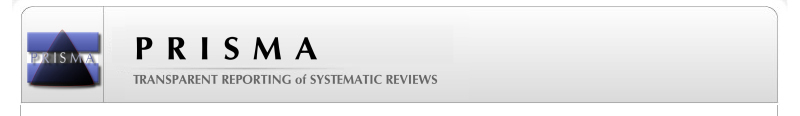
**PRISMA 2009 Flow Diagram**

**Screening**

**Included**

**Eligibility**

**Identification**

Records identified through database searching
(n = 1027)

Additional records identified through other sources
(n = 0)

Records after duplicates removed
(n = 1027 )

Records screened
(n = 1027 )

Records excluded
(n = 841)

Full-text articles assessed for eligibility
(n = 186)

Full-text articles excluded, with reasons
(n = 172)

Did not fulfill criteria for credibility (n=172)

Studies included in qualitative synthesis
(n = 14)
